# The relationship between physical exercise and learning engagement among chinese college students: the chain mediating roles of cognitive flexibility and psychological resilience

**DOI:** 10.3389/fpsyg.2026.1731426

**Published:** 2026-01-21

**Authors:** Zigu Zhang, Zhijian Rao, Mingyang Liu, Jin Han, Lifang Zheng

**Affiliations:** 1 College of Physical Education, Shanghai University, Shanghai, China; 2 College of Physical Education, Shanghai Normal University, Shanghai, China; 3 Exercise Biological Center, China Institute of Sport Science, Beijing, China

**Keywords:** cognitive flexibility, college students, learning engagement, physical exercise, psychological resilience

## Abstract

**Objectives:**

This study tested a sequential mediation model examining whether cognitive flexibility and psychological resilience explain the relationship between physical exercise and learning engagement among Chinese college students.

**Methods:**

A cross-sectional study was conducted with 670 Chinese college student. Participants completed the Physical Activity Rating Scale, Learning Engagement Scale, Cognitive Flexibility Inventory, and Psychological Resilience Scale. Data were analyzed using SPSS 27.0 and the PROCESS macro (Version 4.2) for serial mediation analysis with 5,000 bootstrap samples.

**Results:**

Regression analyses revealed that physical exercise positively predicted cognitive flexibility (β = 0.507, *p* < 0.001) and psychological resilience (β = 0.381, *p* < 0.001). Cognitive flexibility further predicted psychological resilience (β = 0.336, *p* < 0.001) and learning engagement (β = 0.370, *p* < 0.001). Crucially, the mediation analysis confirmed a significant direct effect of exercise on learning engagement (β = 0.143, 95% CI = [0.068, 0.218]). Three specific indirect effects were significant: through cognitive flexibility (β = 0.188, 95% CI [0.141, 0.234]), through psychological resilience (β = 0.086, 95% CI [0.050, 0.126]), and serially through both (β = 0.038, 95% CI [0.020, 0.059]).

**Conclusion:**

This cross-sectional study reveals a significant positive association between physical exercise and learning engagement among Chinese college students. The findings support a model in which cognitive flexibility and psychological resilience act as sequential mediators in this relationship. These results suggest that fostering psychological resources alongside physical activity may be a relevant consideration for promoting student engagement in learning. Future longitudinal and intervention studies are needed to establish causality.

## 1 Introduction

Learning engagement, a positive and fulfilling psychological state characterized by strong identification with sustained concentration in learning activities, is a crucial indicator of students’ participation in the learning process and a key measure of higher education quality (Fredricks et al., 2004; Doo and Kim, 2024). It significantly promotes academic achievement, with higher learning engagement linked to greater motivation, active participation, and better outcomes (Kahu, 2013; Shao et al., 2024). Conversely, low learning engagement is associated with academic struggles, anxiety and increased dropout risk (Kim et al., 2019). Despite its importance, prevalent issues like academic procrastination and lack of motivation represent major bottlenecks to educational quality (Zhou and Ahmad, 2025). Therefore, identifying effective pathways to enhance college students’ learning engagement is of substantial theoretical and practical significance.

Among various influencing factors, physical exercise emerges as a potentially significant predictor. Beyond its physical health benefits, exercise enhances cognitive performance and has been positively linked to academic achievement (Du et al., 2023; Ericsson and Karlsson, 2014). Consistent with this, research on Chinese university students confirms a positive correlation between physical exercise, self-efficacy and learning engagement (Gao et al., 2025). However, the underlying psychological mechanisms through which physical exercise influences learning engagement remain inadequately explained, limiting the development of targeted interventions.

While the positive role of physical exercise is established, the specific pathways require clarification. Cognitive flexibility and psychological resilience are two key psychological factors critical for learning engagement (Masten et al., 2022; Zhao et al., 2024). Cognitive flexibility enables students to adapt to new learning demands, thereby improving focus and efficiency (Bukowski et al., 2024), and evidence shows it can be enhanced by physical activity across different populations (Lerche et al., 2018; Manzari Tavakoli et al., 2024; Tseng et al., 2022). Similarly, psychological resilience helps students manage academic pressures and maintain learning motivation (Bagdiūnien et al., 2025), and it is strongly correlated with regular physical exercise (Peng et al., 2025; Xiu, 2014). Despite this, previous studies have not sufficiently examined the integrated mediating roles of cognitive flexibility and psychological resilience in connecting physical exercise to learning engagement. A clear rationale for how exercise translates into learning engagement through these specific psychological capacities is lacking.

To address this gap, the present study aims to explicitly examine the mediating roles of cognitive flexibility and psychological resilience in the relationship between physical exercise and learning engagement among Chinese college students. We hypothesize that physical exercise will be positively associated with learning engagement, and that this relationship will be sequentially mediated by cognitive flexibility and psychological resilience. By testing this model, the study seeks to provide a clearer mechanistic explanation and robust empirical foundation for designing holistic interventions that promote both mental wellbeing and leaning engagement through physical activity.

## 2 Theoretical basis and hypothesis

### 2.1 The relationship between physical exercise and learning engagement

**FIGURE 1:**
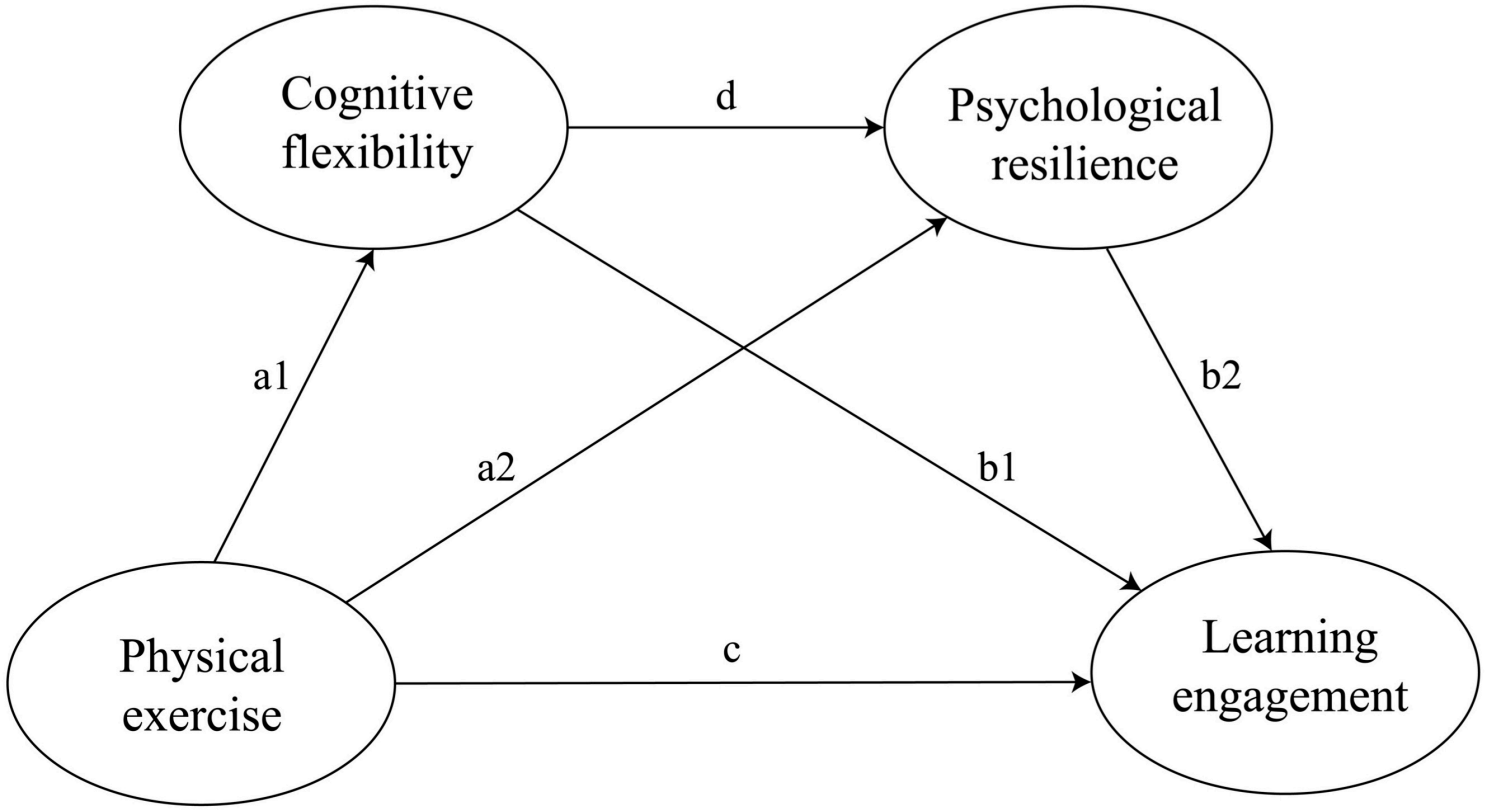
A hypothesis chain mediation model of physical exercise and learning engagement.

Physical exercise is defined as planned physical activity of a certain intensity, frequency, and duration, aim at enhancing physical and mental health (Pan et al., 2021). Learning engagement, or academic engagement, refers to a positive and fulfilling cognitive-emotional state displayed by students during the learning, typically characterized by three dimensions: vigor, dedication, and absorption (Schaufeli W.B et al., 2002). Vigor reflects high energy and psychological resilience in learning; dedication refers to a sense of enthusiasm and meaningful involvement in academic activities; and absorption denotes full concentration on learning tasks with minimal distraction (Carmona-Halty et al., 2021). As a key predictor of academic achievement (Sahni, 2023), greater learning engagement is generally associated with better learning outcomes and performance (Yildiz Durak et al., 2025). Empirical evidence supports the role of physical exercise in promoting learning engagement. For example, Kotaman and Evran (2021) demonstrated that incorporating brief physical activity into class time can enhance college students’ motivation and academic performance. Analysis of data from the China Education Panel Studies (CEPS) also confirms the positive effect of physical exercise on adolescents’ academic outcomes (Ren et al., 2021). Randomized controlled trials further indicate that physical exercise can improve concentration among primary school students (Spitzer and Hollmann, 2013), while low-intensity aerobic activity has been shown to increase vitality and reduce fatigue, thereby boosting college students’ classroom motivation (Tamura et al., 2022). Estrada-Tenorio et al. (2020) further noted that students with higher levels of physical activity tend to exhibit greater self-discipline and focus. At the mechanistic level, exercise self-efficacy and flow experience during physical activity have been identified as factors that foster deeper engagement in learning (Huang et al., 2025). Based on the theoretical and empirical evidence outlined above, this study proposes the following hypothesis:

Hypothesis 1: Physical exercise positively predicts learning engagement among college students (as shown in Figure 1, path c).

### 2.2 The mediating role of cognitive flexibility

Cognitive flexibility, a core component of executive function, refers to the ability to adapt one’s thinking and behavior in response to changing environmental and task demands (Shukla et al., 2020). Greater cognitive flexibility is generally associated with faster learning, stronger problem-solving skills, and better situational adaptation. Substantial evidence indicates that physical exercise promotes a range of cognitive functions, including executive control, working memory, and cognitive flexibility (Chen C. et al., 2022; Liu et al., 2025; Zhu et al., 2020). Notably, both acute and chronic exercise have been shown to enhance cognitive flexibility. For instance, a single 20-min session of moderate-intensity aerobic exercise can lead to immediate improvements in cognitive flexibility, with effects sustained for 30–60 min post-exercise (Shukla et al., 2020; Shukla and Heath, 2022). Among youth people, both high-intensity interval training (HIIT) and moderate-intensity continuous exercise (MICE) have demonstrated positive effects on cognitive flexibility, especially after accounting for baseline performance (Liu et al., 2025). Longer-term interventions, such as a 10-week physical activity program during school breaks, have also been effective in improving cognitive flexibility in primary school students (Pedro Ángel et al., 2021). Exercise-induced benefits in cognitive flexibility extend to clinical and subclinical populations as well. For instance, Exercise frequencies has been significantly correlated with cognitive flexibility in individuals with anxiety disorders (Riho and Anu, 2024). A 12 weeks physical activity intervention significantly improved cognitive flexibility in children with internet addiction (Tseng et al., 2022), and a boxing training program was found to reduce cognitive rigidity and enhance task-switching ability among high school students with depressive tendencies (Hao, 2024). Together, these findings offer robust empirical support for the role of physical exercise in promoting cognitive flexibility across different populations and intervention formats.

Cognitive flexibility plays a vital role in the learning (Shah, 2025). Students with greater cognitive flexibility are better able to shift between concepts, adapt to new learning environments and instructional methods, and use creative strategies to overcome academic challenges (Sugilar et al., 2022). This is especially relevant in the post-pandemic era, as students increasingly rely on cognitive flexibility to navigate online and blended learning environments (Shah, 2025). Those with greater cognitive flexibility tend to engage as active participants rather than passive recipients in learning, which in turn enhances their learning engagement (Orakcı, 2021). Empirical studies further support a significant positive correlation between cognitive flexibility and learning engagement. Generally, the more adaptable and flexible a student’s thinking, the greater their level of learning engagement tends to be. For example, Zhao et al. (2024) found in a study of Chinese college students that psychological resilience indirectly influenced learning engagement through a chain mediation involving goal orientation and cognitive flexibility. Such findings reinforce that cognitive flexibility is a positive predictor of learning engagement. Based on the theoretical and empirical literature reviewed, this study proposes the following hypothesis:

Hypothesis 2: Cognitive flexibility mediates the relationship between physical exercise and learning engagement (as shown in Figure 1, path a_1_→b_1_).

### 2.3 The mediating role of psychological resilience

Students’ learning engagement is influenced by a variety of factors, with psychological resilience serving as a key psychological resource (Yang et al., 2024). According to psychological resilience theory, the capacity to thrive in the face of adversity is critical for academic performance (Masten et al., 1990). Psychological resilience is defined as an individual’s ability to maintain or quickly restore mental wellbeing when confronted with stress or trauma, and to continue developing in a positive, adaptive manner (Arpaci and Gundogan, 2022; Fletcher and Sarkar, 2013). As a form of self-regulation, it helps individuals cope effectively with challenging and has been shown to positively influence learning engagement. Empirical studies indicate that psychological resilience significantly and positively predicts learning engagement (Romano et al., 2021). Students with higher psychological resilience are better able to sustain positive emotions, manage academic stress, and persist in pursuing learning goals despite difficulties (Yang et al., 2024). Wang et al. (2025) demonstrated that psychological resilience not only directly affects college students’ online learning performance and satisfaction, but also mediates the relationship through reduced academic burnout. Research on middle-school students further confirms that psychological resilience indirectly promotes physical activity participation through enhanced learning motivation, underscoring its broad role in learning-related behaviors (Hu H. et al., 2025). Additionally, academic self-efficacy has been found to influence learning engagement via psychological resilience (Wang and Zhang, 2024), suggesting that individuals with higher psychological resilient tend to exhibit more positive learning emotions, optimistic task attitudes, and stronger self-confidence—all of which facilitate more proactive learning behaviors and ultimately improve engagement.

Physical exercise has gained substantial empirical support as an effective means of enhancing psychological resilience. Studies indicate that physical exercise can significantly improve an individual’s psychological resilience, thereby strengthening their ability to cope with academic and life challenges (Bai et al., 2022). For example, research on Chinese university students has shown that the intensity, frequency, and duration of physical exercise are positively correlated with positive coping styles, with psychological resilience mediating this relationship (Zhang J. et al., 2024). Other work suggested that physical exercise boosts psychological resilience by enhancing physical self-esteem (Hu W. et al., 2025). From a neurobiological perspective, regular exercise promotes neural plasticity and optimizes brain structure and function, which in turn improves stress resilience (Jiang et al., 2025). Furthermore, physical exercise alleviates negative emotions such as anxiety and depression among college students through the mediating role of psychological resilience (Adamek et al., 2025; Liu et al., 2023; Zuo et al., 2025). As negative emotions are reduced, students can devote more cognitive and emotional resources to learning, thereby enhancing their engagement (Rowe and Fitness, 2018). Another line of research demonstrates that the protective effect of physical exercise against academic burnout is mediated sequentially by self-efficacy and psychological resilience (Chen K. et al., 2022). In summary, psychological resilience not only helps students maintain emotional stability and motivation in stressful environments but also facilitates learning engagement through multiple psychosocial mechanisms, thereby supporting better academic performance. Based on the theoretical and empirical literature reviewed, this study proposes the following hypothesis:

Hypothesis 3: Psychological resilience mediates the relationship between physical exercise and learning engagement (as shown in Figure 1, path a_2_→b_2_).

### 2.4 The chain mediation of cognitive flexibility and psychological resilience

Cognitive flexibility contributes to learning engagement not only directly but also indirectly by fostering psychological resilience. Previous studies indicate that cognitive flexibility is positively associated with mental health and wellbeing, while being negatively correlated with psychological problems such as depression (Murphy et al., 2012) and anxiety (Han et al., 2011). Furthermore, cognitive flexibility has been shown to exert a positive influence on the psychological resilience of college students. Research suggests a positive correlation between cognitive control and cognitive flexibility, with higher cognitive flexibility contributing to greater psychological resilience (Bacolu and Kocabyk, 2025). During the COVID-19 pandemic, students with low perceived pressure and high cognitive flexibility demonstrated stronger psychological resilience in the face of traumatic experiences (Luo et al., 2022). Additionally, individuals with greater cognitive flexibility are better able to consider situations from multiple perspectives, mitigate the interference of negative emotions, and enhance their capacity to cope with stress (Arici-Ozcan et al., 2019). When people can flexibly adjust their cognitive strategies, they are more likely to perceive challenges as opportunities for learning and growth, thereby further strengthening psychological resilience. Accordingly, this study propose the following hypothesis:

Hypothesis 4: Cognitive flexibility and psychological resilience play a chain mediating role in the relationship between physical exercise and learning engagement (as shown in Figure 1, path a_1_→d→b_1_).

This study investigates the relationship between physical exercise and learning engagement among college students, together with the mediating roles of cognitive flexibility and psychological resilience. Based on the above hypotheses, a chain mediation model is proposed (as shown in Figure 1).

## 3 Materials and methods

### 3.1 Study design and participants

This study adopted a cross-sectional, correlational survey design to examine the relationships between physical exercise, learning engagement, cognitive flexibility, and psychological resilience among university students. Participants were recruited from a comprehensive university in Shanghai using a mixed sampling strategy. This strategy combined convenience sampling with stratified and cluster sampling based on academic year and major category to enhance sample diversity and representativeness. The required sample size was estimated using G*Power 3.1 software. For a multiple regression analysis with four predictors, a medium effect size (f^2^ = 0.15), α = 0.05, and power = 0.95, the minimum sample size was calculated to be 129. A total of 700 questionnaires were distributed, and all were returned. After excluding invalid responses (e.g., inconsistent answer patterns or excessively short completion times), 670 valid questionnaires were retained, yielding an effective response rate of 95%. Participant demographics are summarized in Table 1. The sample consisted of 343 males (51.19%) and 327 females (48.81%), with a mean age of 20.09 years. In terms of major distribution, 485 participants (72.4%) were from science and engineering disciplines, 129 (19.2%) from humanities and social sciences, and 56 (8.4%) from arts. Regarding family background, 337 participants (50.30%) were from one-child families, while 333 (49.70%) had siblings. Additionally, 110 participants (16.42%) were from rural areas, and 560 (83.58%) were from urban areas.

**TABLE 1:** Distribution of demographic variables among survey participants.

Category	Number of people	Percentage
	*n*	%
**Gender**		
Male	343	51.19
Female	327	48.81
**Major**		
Science and engineering	485	72.4
Humanities and social sciences	129	19.2
Arts	56	8.4
**Registered residence**		
Rural areas	110	16.42
Urban areas	560	83.58
**Only child family**		
Yes	337	50.30
No	333	49.70

### 3.2 Procedure and ethical considerations

Before participation, all students received standardized written and oral instructions explaining the study’s purpose, voluntary nature, anonymity, and their right to withdraw at any time. Informed consent was obtained from all participant. The study was approved by the Ethics Committee of Shanghai University (Approval No. ECSHU 2025-054). Data were collected during scheduled class sessions to ensure a consistent administration environment.

### 3.3 Research tools

#### 3.3.1 Physical Activity Rating Scale (PARS-3)

Physical exercise was assessed using the revised Physical Activity Rating Scale (PARS-3) developed by Liang (1994). The scale consists of three items measuring exercise intensity, frequency, and duration per session, each rated on a five-point scale. A composite score (range 0–100) is calculated as: intensity × (duration−1) × frequency. Higher scores indicate higher physical activity levels. The scale has demonstrated good reliability and validity in previous Chinese student samples. In this study, Cronbach’s α was 0.830.

#### 3.3.2 Utrecht Work Engagement Scale for Students (UWES-S)

Learning engagement was measured using the Utrecht Work Engagement Scale for Students (UWES-S). The original scale was developed by Schaufeli W.B et al. (2002) and subsequently translated and validated in Chinese by Fang et al. (2008). The scale includes 17 items across three subscales: vigor (6 items), dedication (5 items), and absorption (6 items). Items are rated on a five-point Likert scale (1 = “never,” to 5 = “always”). The total score ranges from 17 to 85, with higher scores indicating greater level of learning engagement. The scale demonstrated excellent internal consistency in this study, with a Cronbach’s α of 0.922.

#### 3.3.3 Cognitive Flexibility Scale

Cognitive flexibility was assessed using the Chinese version of the Cognitive Flexibility Scale. This scale was originally developed by Dennis and Vander Wal (2010) and was subsequently revised and validated for Chinese populations by Liu C. et al. (2024) The scale comprises 20 items across two dimensions: selectivity (12 items) and controllability (8 items). Responses are given on a five-point scale (1 = “never” to 5 = “always”). The total score ranges from 20 to 100, with higher scores reflecting greater cognitive flexibility. The scale showed excellent reliability in this study, with a Cronbach’s α of 0.957.

#### 3.3.4 Psychological resilience scale

Psychological resilience was measured using the Chinese version of the 25-item Connor-Davidson Resilience Scale (CD-RISC), which was originally developed by Connor and Davidson (2003) and later validated in Chinese populations by Xiaonan and Jianxin (2007). The scale consists of 25 items across three dimensions: tenacity (13 items), strength (8 items), and optimism (4 items). Items are rated on a five-point Likert scale (1 = “never” to 5 = “almost always”). The total score ranges from 25 to 125, with higher scores indicating greater psychological resilience. In the present study, the scale exhibited excellent internal consistency, with a Cronbach’s α of 0.922.

### 3.4 Statistical analysis

Data were analyzed using SPSS 27.0 and the PROCESS macro (Version 4.2). First, descriptive statistics (means and standard deviations) were calculated for all main variables. The internal consistency for each scale was assessed using Cronbach’s α. To examine common method bias, Harman’s single-factor test was conducted. The unrotated factor solution showed that the maximum variance explained by a single factor was 33.81%, which is below the commonly recommended threshold of 40%, suggesting that common method bias was not a serious concern in this study. Pearson correlation analysis was used to assess the relationships among physical exercise, learning engagement, cognitive flexibility, and psychological resilience. To test the hypothesized serial mediation model, we used Model 6 of the SPSS PROCESS macro with 5,000 bootstrap resamples. The model examined whether cognitive flexibility and psychological resilience sequentially mediated the relationship between physical exercise and learning engagement. Prior to the analysis, preliminary checks were conducted and confirmed that the assumptions of linearity, normality, and homoscedasticity were met. The significance of direct and indirect effects was determined using bias-corrected 95% bootstrap confidence intervals; effects were considered statistically significant if the confidence interval did not include zero.

## 4 Results

### 4.1 Common method bias test

As all data were collected via self-report, Harman’s single-factor test was conducted as a preliminary check for common method variance. The unrotated exploratory factor analysis extracted nine factors with eigenvalues greater than 1, with the first factor accounting for 33.813% of the variance, below the conventional threshold of 40% (Podsakoff et al., 2003; Zhou and Long, 2004). This indicates that common method bias was not a serious concern in the present study.

### 4.2 Descriptive statistics and correlations

Means, standard deviations (SD), and intercorrelations for all main variables are presented in Table 2. Physical exercise was significantly and positively correlated with cognitive flexibility (*r* = 0.516, *p* < 0.01), psychological resilience (*r* = 0.572, *p* < 0.01) and learning engagement (*r* = 0.467, *p* < 0.01) underwent Pearson bivariate correlation analysis. Cognitive flexibility and psychological resilience were significantly and positively correlated with learning engagement (*r* = 0.574, *r* = 0.521, *p* < 0.01). Additionally, there is a significantly correlation between psychological resilience and cognitive flexibility (*r* = 0.558, *P* < 0.01), indicating suitable conditions for subsequent mediation analysis.

**TABLE 2:** Correlation analysis of physical exercise, cognitive flexibility, psychological resilience and learning engagement.

Variables	*M*	SD	Physical exercise	Cognitive flexibility	Psychological resilience	Learning engagement
Physical exercise	19.82	27.78	–	–	–	–
Cognitive flexibility	3.20	0.97	0.516***	–	–	–
Psychological resilience	3.33	1.70	0.572***	0.558***	–	–
Learning engagement	2.94	0.89	0.467***	0.574***	0.521***	–

*N* = 670,

***p < 0.001.

### 4.3 Mediating effect test

A chain mediation analysis was conducted to examine whether cognitive flexibility and psychological resilience sequentially mediate the relationship between physical exercise (independent variable) and learning engagement (dependent variable). The hypothesized model is presented in Figure 1.

First, a series of regression analyses (controlling for gender and age) were performed to establish the foundational paths (Table 3). Physical exercise significantly and positively predicted cognitive flexibility (β = 0.507, *p* < 0.001, 95% CI [0.442, 0.572]) and psychological resilience (β = 0.381, *p* < 0.001, 95% CI [0.317, 0.449]). Furthermore, cognitive flexibility, in turn, was a significant positive predictor of psychological resilience (β = 0.336, *p* < 0.001, 95% CI [0.267, 0.400]) and learning engagement (β = 0.370, *p* < 0.001, 95% CI [0.297, 0.445]). Psychological resilience also positively predicted learning engagement (β = 0.224, *p* < 0.001, 95% CI [0.146, 0.305]). When the mediators were included, the direct effect of physical exercise on learning engagement remained significant but was reduced (β = 0.143, *p* < 0.001, 95% CI [0.068, 0.218]), suggesting partial mediation.

**TABLE 3:** Regression analysis results between variables.

Variable	Predictor	β	95% CI (β)	t	*P*	R^2^	F
Cognitive flexibility	Gender	−0.096	[−0.163, −0.032]	−2.827	0.005	0.280	84.701 (3,666)***
	Age	0.050	[−0.068, 0.062]	0.855	0.393		
	Physical exercise	0.507	[0.442, 0.572]	15.225	<0.0001		
Psychological resilience	Gender	−0.183	[−0.237, 0.123]	−6.173	<0.0001	0.455	137.685 (4,665)***
	Age	−0.022	[−0.042, 0.071]	−0.424	0.672		
	Physical exercise	0.381	[0.317, 0.449]	11.310	<0.0001		
	Cognitive flexibility	0.336	[0.267, 0.400]	9.928	<0.0001		
Learning engagement	Gender	−0.024	[−0.081, 0.041]	−0.771	0.441	0.407	90.082 (5,664)***
	Age	0.049	[−0.006, 0.112]	0.910	0.363		
	Physical exercise	0.143	[0.068, 0.218]	3.721	<0.0001		
	Cognitive flexibility	0.370	[0.297, 0.445]	9.779	<0.0001		
	Psychological resilience	0.224	[0.146, 0.305]	5.522	<0.0001		

CI, confidence interval.

***p < 0.001.

**FIGURE 2:**
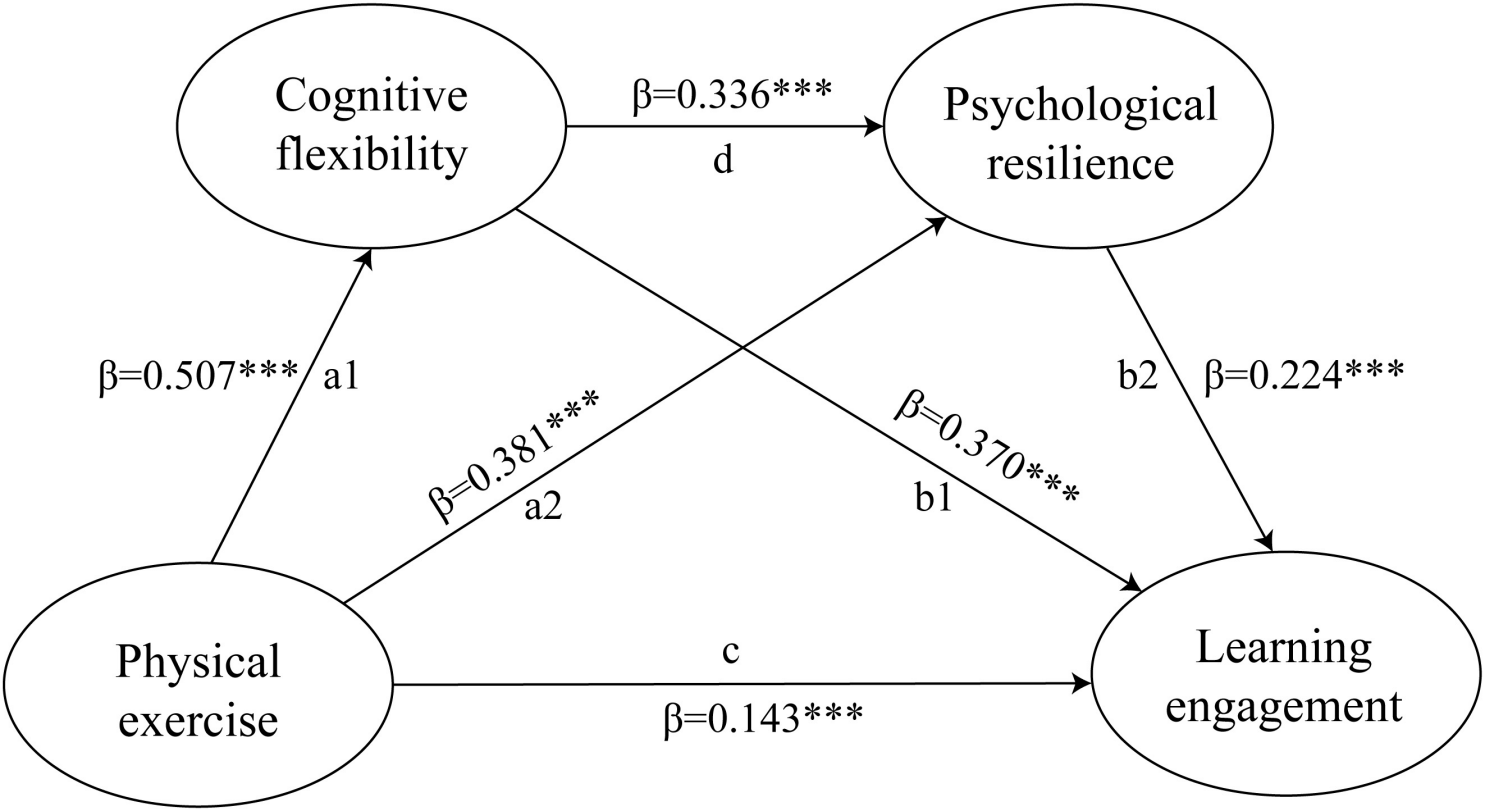
The serial mediation model with cognitive flexibility and psychological resilience as mediators of the linkage between physical exercise and learning engagement. ***p < 0.001.

The significance of the indirect effects was tested using bias-corrected bootstrapping with 5,000 resamples. The results, summarized in Table 4 and visualized in Figure 2, revealed a significant total effect of physical exercise on learning engagement (effect = 0.456, 95% CI = [0.389, 0.523]). The direct effect was also significant (effect = 0.143, 95% CI = [0.068, 0.218]), accounting for 31% of the total effect. The bootstrapping analysis confirmed significant indirect effects for all three hypothesized mediating pathways. A significant total indirect effect was observed, indicating that the specified mediators collectively accounted for a substantial portion of the association. Specifically, the mediation via cognitive flexibility alone was strongest (effect = 0.188, 95% CI [0.141, 0.234]), explaining 42% of the total effect. The pathway through psychological resilience alone was also significant, albeit with a smaller magnitude (effect = 0.086, 95% CI [0.050, 0.126]; 19% of the total effect). Furthermore, the hypothesized sequential chain via cognitive flexibility and then psychological resilience was statistically significant (effect = 0.038, 95% CI [0.020, 0.059]), accounting for an additional 8% of the total effect. These findings indicate that physical exercise is associated with higher learning engagement both directly and indirectly through three significant pathways. The strongest mechanism is the mediation via cognitive flexibility alone. Furthermore, the significant chain mediation effect supports our hypothesis that physical exercise is linked to increased cognitive flexibility, which is associated with greater psychological resilience, ultimately contributing to enhanced learning engagement.

**TABLE 4:** Chain mediation analysis of the impact of physical exercise on learning engagement.

Pathway	Effect	Standardized effect	Boot SE	95%CIBOOT LLCI	95%CI boot ULCI	Relative effect proportion
**Direct effects**						
Physical exercise → learning engagement	0.078	0.143	0.38	0.068	0.218	31%
**Indirect effects**						
Physical exercise → cognitive flexibility → learning engagement	0.103	0.188	0.24	0.141	0.234	42%
Physical exercise → psychological resilience → learning engagement	0.047	0.086	0.19	0.050	0.126	19%
Physical exercise → cognitive flexibility → psychological resilience → learning engagement	0.021	0.038	0.10	0.020	0.059	8%
**Total effects**						
Physical exercise → learning engagement	0.248	0.456	0.34	0.389	0.523	100%

## 5 Discussion

This cross-sectional study examined the psychological mechanisms linking physical exercise to learning engagement among Chinese college students, testing a chain mediation model via cognitive flexibility and psychological resilience. The results supported all hypotheses, revealing significant positive associations between physical exercise and learning engagement. Crucially, mediation analyses confirmed three significant indirect pathways: through cognitive flexibility alone, through psychological resilience alone, and sequentially through both mediators (cognitive flexibility → psychological resilience). These findings suggest that the benefits of physical exercise for learning engagement are not merely direct but are substantially channeled through enhanced cognitive and emotional-resource capacities.

### 5.1 Association between physical exercise and learning engagement

The results of this study indicate a significant positive association between physical exercise and learning engagement among college students. This finding supports Hypothesis 1, suggesting that students who engage in higher levels of physical activity tend to demonstrate greater learning engagement. This result is consistent with previous research (Du et al., 2023; Gao et al., 2025), further supporting the observed link between physical exercise and academic involvement in the literature.

Existing theoretical and empirical work proposes several mechanisms that could explain this link. Research suggests that physical exercise may enhance students’ learning engagement by improving academic performance and concentration (Chawre, 2025; Zarazaga-Peláez et al., 2024), and by helping regulate emotional states, thereby potentially increasing learning efficiency (Qin et al., 2024). Furthermore, physical exercise is thought to strengthen self-efficacy, which could enable students to maintain confidence and a positive attitude toward learning, in turn promoting active participation (Gao et al., 2025). From a neurophysiological perspective, physical activity is associated with increased cerebral blood flow, neurogenesis, and enhancements in memory, attention, and executive function—processes that are believed to support more efficient learning (Lehmann et al., 2020; Zhao, 2024). Neuroimaging studies indicate that exercise activates key brain regions such as the prefrontal cortex, parietal lobe, and posterior cingulate cortex, which are critically involved in executive control, attention regulation, and information integration (Hillman et al., 2008). In addition, exercise-induced changes in neurotransmitters such as dopamine and norepinephrine modulate the brain’s rewards and attention systems, facilitating memory encoding and retrieval (Alizadeh Pahlavani, 2024). As noted by Vaynman et al. (2004), exercise supports cognitive function by enhancing synaptic plasticity and promoting neurotransmitter release. These proposed mechanisms collectively provide a plausible theoretical basis for understanding how physical exercise might be linked to improved learning engagement. In summary, the association observed in the present study aligns with these multidisciplinary perspectives, corroborating the positive link between physical exercise and learning engagement among college students reported in the literature.

### 5.2 Cognitive flexibility as a mediator between physical exercise and learning engagement

The present study revealed a significant indirect effect of physical exercise on learning engagement via cognitive flexibility, accounting 42% of the total effect, which supports Hypothesis H2. The observed pattern is consistent with a process in which physical exercise is associated with higher cognitive flexibility, which in turn is associated with greater learning engagement. These findings align with previous research reporting positive correlations between physical exercise and cognitive flexibility, suggesting that students who engage regularly in physical activity tend to demonstrate greater cognitive adaptability (Mou et al., 2023; Shukla et al., 2020). Furthermore, existing studies have reported a significant positive association between cognitive flexibility and learning engagement (Tseng et al., 2020; Zhao et al., 2024), indicating that students with more adaptable and flexible thinking patterns generally exhibit higher learning engagement.

Multiple empirical studies support the positive effects of physical exercise on executive functions, including cognitive flexibility (Mou et al., 2023; Shukla et al., 2020). For instance, Shukla et al. (2020) found that even a single session of moderate-intensity aerobic exercise significantly improved an individual’s cognitive flexibility. The potential mechanisms through which physical exercise on cognitive flexibility may involve multiple physiological mechanisms. Physical exercise can influence neurocognitive function by modulating the release and activity of neurotransmitters such as dopamine (Berse et al., 2015). Additionally, aerobic exercise has been shown to enhance synaptic plasticity, inhibit neuronal apoptosis, and help maintain brain homeostasis, which are linked to improved overall brain function (Bettio et al., 2019; Won et al., 2021). From a cognitive neuroscience perspective, this provides a plausible basis for how physical exercise could be connected to learning engagement through gains in cognitive flexibility.

The present study’s finding—that cognitive flexibility is positively associated with learning engagement—also aligns with prior research. Cognitive flexibility has been identified as a mediator between individual traits and learning engagement, as it may help students better adapt to changes and challenges in academic settings, thereby potentially facilitating greater learning engagement (Zhao et al., 2024). Similarly, Bayley (2024). emphasized the critical role of cognitive flexibility in dynamic situations, noting that this ability enables individuals to solve problems more effectively and adapt to new environments. This perspective aligns with the pattern observed in the current study, wherein the association between physical exercise and learning engagement appears to involve cognitive flexibility—a capacity linked to task switching, multi-perspective thinking, and adaptive problem-solving, all of which are conducive to sustained learning engagement (Achdiyah et al., 2023). Taken together, these results highlight cognitive flexibility as a significant mediator in the relationship between physical exercise and learning engagement among Chinese college students.

### 5.3 Psychological resilience as a mediator between physical exercise and learning engagement

The results of this study indicate significant positive associations between physical exercise and psychological resilience, as well as between psychological resilience and learning engagement. Furthermore, the mediation analysis revealed a significant indirect effect of physical exercise on learning engagement through psychological resilience, which supports Hypothesis H3. These findings are consistent with previous research reporting a positive link between physical exercise and psychological resilience among college students (Dunston et al., 2022; San Román-Mata et al., 2020). Regular physical activity is associated not only with improved physical fitness but also with better emotional regulation and stress-coping abilities, which linked to the development of psychological resilience (Xu et al., 2021).

As an important adaptive psychological resource, psychological resilience is connected to an individual’s ability to maintain a positive sense of self-efficacy when facing challenges, thereby potentially enhancing their capacity to cope with difficulties (Peng et al., 2025). In line with the conservation of resources theory (Hobfoll, 2002), individuals with richer psychological resources are more likely to exhibit positive behavioral outcomes. Psychological resilience, which reflects one’s ability to adapt and grow under adversity (Fletcher and Sarkar, 2013; Romano et al., 2019), enables students to better adjust to the academic environment, manage learning-related pressures, and sustain higher learning motivation (Connor and Davidson, 2003; Fernández-Castillo and Fernández-Prados, 2021). Moreover, the present study found that psychological resilience was positively associated with learning engagement, corroborating earlier findings (Wang and Zhang, 2024). Students with higher psychological resilience also tend to experience more pleasure and persistence in learning, along with clearer academic goals, greater vitality, and stronger focus (Ang et al., 2024) —all of which are correlates of higher learning engagement. In summary, the pattern of results suggests that physical exercise is indirectly linked to college students’ learning engagement via its association with psychological resilience. This cross-sectional evidence is consistent with a model in which psychological resilience serves as a mediating mechanism in this relationship.

### 5.4 A chain mediating role of cognitive flexibility and psychological resilience in the relationship between physical exercise and learning engagement

The primary aim of this study was to investigate the potential chain mediation roles of cognitive flexibility and psychological resilience in the relationship between physical exercise and learning engagement. The results supported our hypothesized model, revealing a significant total effect of physical exercise on learning engagement. Notably, while a direct effect was observed, the majority (69%) of this influence was explained by the indirect pathways, underscoring the critical role of the examined psychological mechanisms.

The results of the chain mediation analysis provide nuanced insights into how physical exercise influences learning engagement. Notably, the indirect effects, collectively explaining 69% of the total effect, underscore the primacy of psychological mechanisms over direct influence. The strongest mediator was cognitive flexibility (indirect effect β = 0.188), accounting for 42% of the total effect. This suggests that a primary benefit of physical activity for learning may be its capacity to enhance mental agility and the ability to switch between tasks or concepts. Psychological resilience also played a meaningful, though comparatively smaller, independent mediating role (β = 0.086). Importantly, the significant serial pathway (Physical exercise → Cognitive flexibility → Psychological resilience → Learning engagement, β = 0.038) reveals a more complex dynamic: improvements in cognitive flexibility may also foster greater psychological resilience, creating a synergistic effect that further promotes engagement in learning. These findings position cognitive flexibility as a central and potentially primary mechanism, while highlighting psychological resilience as both a complementary and sequentially-influenced factor. Although the absence of direct precedent for the identical parallel-serial model in the literature limits direct comparisons, the magnitude of our effects is consistent with the range of significant indirect effects commonly reported in broader research examining how distal factors influence outcomes through proximal psychological mechanisms. For instance, studies investigating how physical activity influence behavioral and adaptive outcomes via psychological mechanisms such as self-efficacy or positive affect often report standardized indirect effects within a comparable range (β = 0.013–0.22) (Leng et al., 2025; Liu L. et al., 2024; Zhang G. et al., 2024; Zhang, 2025). This supports the stable and non-negligible explanatory power of the psychological pathways identified in our study.

These observed mechanisms align with and extend several theoretical perspectives. Studies have reported a positive association between cognitive flexibility and psychological resilience (Ceken, 2025; Nakhostin-Khayyat et al., 2024). Behaviorally, cognitive flexibility, the ability to adapt cognitive processing strategies in response to new or changing demands, enables individuals to appraise stressors from multiple perspectives and shift away from rigid, negative thought patterns. This capacity is directly linked to more adaptive stress coping and reduced vulnerability to anxiety and depression (Arici-Ozcan et al., 2019; Han et al., 2011), thereby building psychological resilience. Empirical studies support this link, showing that cognitive flexibility is a significant positive predictor of psychological resilience among various populations, including university students (Bacolu and Kocabyk, 2025; Luo et al., 2022). The neuropsychological mechanisms underlying this transformation provide deeper justification for the pathway. Cognitive flexibility is fundamentally supported by executive control networks centered in the prefrontal cortex (PFC), including the dorsolateral (dlPFC), orbitofrontal (OFC), and ventromedial (vmPFC) regions (Bo et al., 2024; Hiser and Koenigs, 2018). These regions form an integrated “cognitive-emotional” circuit. The dlPFC is crucial for adjusting cognitive strategies, while the OFC and vmPFC are involved in valuing emotional information and integrating cognition with affect (Arnsten and Rubia, 2012). A key mechanism linking cognitive flexibility to psychological resilience is cognitive reappraisal—the flexible reinterpretation of emotional stimuli—which heavily relies on the regulatory influence of the vmPFC and OFC over the amygdala, a core limbic structure for threat and fear processing (Arnsten and Rubia, 2012). Effective reappraisal, a hallmark of cognitive flexibility, involves down-regulating amygdala activity via these PFC pathways. Neurochemically, this top-down regulation is facilitated by neurotransmitters like serotonin (5-HT) and GABA, which enhance vmPFC inhibition over the amygdala (Celada et al., 2013). Therefore, by strengthening PFC-amygdala circuit homeostasis, cognitive flexibility enhances an individual’s capacity for emotion regulation, a core component of psychological resilience. This neurocognitive foundation explains howflexible cognition translates into emotional stability and stress resilience.

Within the later part of this chain, psychological resilience functions as a key psychological resource correlated with learning engagement. College students with higher psychological resilience tend to experience more positive emotions, which are linked to greater initiative and concentration during learning (Yang et al., 2024). According to the conservation of resources theory, positive emotions and psychological resilience, as internal psychological resources, can help individuals recover from stress and sustain effort in academic tasks (Li-xia et al., 2012). Therefore, the cross-sectional data are consistent with a sequential mediation model where physical exercise shows an indirect association with learning engagement through cognitive flexibility and then psychological resilience.

Although our model received support, alternative explanations merit consideration. The observed associations could be influenced by unmeasured third variables. For instance, individuals with higher innate self-regulation or conscientiousness might both exercise more regularly and engage more deeply in learning, potentially inflating the direct association. Similarly, social support or participation in team sports—factors not measured here—could concurrently bolster resilience, cognitive flexibility, and academic motivation. From a contextual standpoint, the specific academic environment (e.g., competitive pressure, online/hybrid learning formats) and type of physical activity (e.g., aerobic vs. mind-body exercises like yoga) may moderate these relationships. Future research should measure and control for such personality traits and social factors, and explore how different exercise modalities and academic contexts shape these psychological pathways.

### 5.5 Limitations and perspectives

While this study provides insights into the mechanisms linking physical exercise to learning engagement, several limitations should be considered when interpreting the findings. First, the cross-sectional design precludes causal inferences. Although the hypothesized mediation model is theoretically grounded, longitudinal or experimental research is needed to establish temporal precedence and causality among the variables. Second, all data were collected via self-report, which may introduce common method variance and social desirability bias. Although procedural remedies (e.g., anonymity, reverse-scored items) were applied and Harman’s single-factor test did not indicate severe bias, the potential for inflated correlations remains. Future studies would benefit from multi-method assessments, such as incorporating objective measures of physical activity (e.g., accelerometers) and peer- or teacher-reported leaning engagement. Third, the sample was drawn from a single university in Shanghai, which may limit the generalizability of findings. Students in different regions of China may vary in their physical exercise habits, academic pressures, and psychological characteristics, influenced by local educational policies, economic development levels, and cultural norms. For instance, students in coastal metropolises like Shanghai might experience distinct lifestyle patterns and academic expectations compared to those in inland or rural areas. Future studies could benefit from recruiting participants from multiple universities across diverse geographic regions (e.g., Eastern, Central, Western China) to enhance the representativeness and external validity of the findings. Fourth, the measurement of physical exercise in this study relies on the PARS-3, a self-reported instrument. Although PARS-3 is widely used and validated in Chinese student populations due to its brevity and practicality, it is not without limitations. The absence of objective monitoring data (e.g., accelerometry, heart-rate-based devices) means that our data may be influenced by subjective perception and potential recall bias. Specifically, participants’ estimates of exercise intensity, duration, and frequency could vary based on individual interpretation and memory accuracy. Future research would benefit from triangulating subjective reports with objective measures to capture physical activity more accurately and to validate the associations found in this study.

## 6 Conclusion

This cross-sectional study aimed to examine the relationship between physical exercise and learning engagement among Chinese university students, as well as the potential mechanism underlying this relationship. The results directly support our hypotheses, confirming that the positive association is not only direct but is significantly mediated by both cognitive flexibility and psychological resilience. Importantly, the findings elucidate a specific chain of influence, wherein physical exercise is linked to greater cognitive flexibility, which in turn is associated with enhanced psychological resilience, ultimately contributing to higher learning engagement.

These conclusions lead to several practical implications. For university athletic departments and coaches, integrating structured physical activity programs into campus life could be advocated not only for health but as an academic enabler. For counselors and sport psychologists, interventions could be dual-focused: promoting physical exercise as a foundation, and simultaneously incorporating cognitive flexibility training (e.g., cognitive reframing, adapting to academic challenges) and psychological resilience-building workshops to amplify the positive effects on student learning engagement. For university administrators and policy makers, creating environments that facilitate regular exercise (e.g., accessible facilities) could be considered a valuable component of holistic student support and academic development strategies.

Future research should address the limitations of this cross-sectional design by employing longitudinal or experimental studies to establish causal relationships. Additionally, investigating the optimal type, frequency, and intensity of physical exercise for cognitive and academic benefits would provide practical guidance. Exploring these mechanisms within specific sub-groups (e.g., by major, or among students with initial learning difficulties) and across different cultural contexts would also enhance the generalizability and precision of the implications.

## Data Availability

The original contributions presented in this study are included in this article/supplementary material, further inquiries can be directed to the corresponding author.
